# Satisfaction Rate After Laser Correction of Presbyopia (Presbyond) Among Patients Aged 40 Years and Older in Taif, Saudi Arabia

**DOI:** 10.7759/cureus.52776

**Published:** 2024-01-23

**Authors:** Talal A Althomali, Abdulrahman N Aloufi, Abdullah A Alharbi, Abdullah H Hafiz, Abdulhamid H Altowairqi, Mohammed H Fattah, Ahmed K Alzahrani, Renad K Althomali, Wehad K Althomali

**Affiliations:** 1 Ophthalmology, Taif University, Taif, SAU; 2 Faculty of Medicine, Taif University, Taif, SAU; 3 Medicine, Dar Al Uloom University, Riyadh, SAU; 4 Medicine, Princess Nourah Bint Abdulrahman University, Riyadh, SAU

**Keywords:** cross-sectional study, refractive surgeries, satisfaction rates, presbyond, presbyopia

## Abstract

Background

Presbyopia is a physiological condition arising due to the loss of accommodation within the crystalline lens. One of the most widely accepted theories of the mechanism of accommodation was that in response to ciliary muscle contraction, the crystalline lens thickness increases, the lens diameter decreases, and both the anterior and posterior curvature of the lens increase, resulting in an increase in lenticular power therefore, accommodation. A contrasting theory suggests that ciliary muscle contraction leads to a selective increase in equatorial zonular tension, with the lens equator moving toward the sclera and the equatorial diameter of the lens increasing. This results in a change of lens optical power. Until now, clinical approaches to correct presbyopia have included monovision, multifocality, and extended depth of focus, all three of which can be achieved surgically on the cornea or by lens surgery.

Methods

This was a cross-sectional study adopted among patients who had Presbyond surgery in Taif City, Saudi Arabia, and were aged 40 years and older. The data was collected by conducting phone interviews to increase the response rate with a prepared questionnaire that was studied to achieve equality between participants to determine whether they were satisfied or not about the results after this surgery. The contact information was retrieved based on hospital records about patients who underwent Presbyond surgery. Data was analyzed using IBM SPSS Statistics for Windows, Version 22 (Released 2013; IBM Corp., Armonk, New York, United States). The data was collected for the period beginning on the first of January 2019 until the first of February 2023.

Results

From the study findings, a significant number of participants (28.1%, n=25) reported experiencing complete improvement and returning to normal life within 1-30 days after surgery. A slightly larger percentage (39.2%, n=35) experienced this within 1-3 months. Most of the participants (80.9%, n=72) reported an overall improvement in their quality of life after the surgery. This included activities such as reading and using a mobile phone. This indicates that the surgery had a positive impact on their daily lives and activities. In terms of recommendations, a total of 49 (55.1%, n=49) participants stated that they were very likely to recommend refractive surgery to a family member or friend experiencing vision problems. The study found that the mean patients’ satisfaction score after one month of surgery is 2.494 units higher than the mean satisfaction score before surgery. This difference was statistically significant (p < .001).

Conclusion

The majority of participants did not experience any problems during the surgery, and most were able to resume their normal activities within a relatively brief period of time. The surgery achieved its goals for the majority of participants, resulting in an improvement in their quality of life. However, some short-term discomfort or adjustment period was reported. Overall, the participants were satisfied with the surgery, with the majority being very likely to recommend it to others. There is room for improvement in addressing concerns such as blurred vision, the need for glasses, and dry eyes. The study also found that patient satisfaction increased significantly after one month of surgery, and the timing of complete improvement and perception of achieving surgical goals were strongly associated with perceived outcomes.

## Introduction

Presbyopia is a physiological condition arising due to the loss of accommodation within the crystalline lens. One of the most widely accepted theories of the mechanism of accommodation was described by Helmholtz as in response to ciliary muscle contraction, the crystalline lens thickness increases, the lens diameter decreases, and both the anterior and posterior curvature of the lens increase, resulting in an increase in lenticular power and, therefore, accommodation [1]. A contrasting theory proposed by Schachar suggests that ciliary muscle contraction leads to a selective increase in equatorial zonular tension, with the lens equator moving toward the sclera and the equatorial diameter of the lens increasing. This results in a change of lens optical power [2]. Therefore, the perfect solution would be to restore this natural process by techniques such as lens refilling, but several challenges need to be addressed and the results are so far limited to experimental models [3,4].

Until now, clinical approaches to correct presbyopia have included monovision, multifocality, and extended depth of focus (EDOF), all three of which can be achieved surgically on the cornea or by lens surgery. However, both monovision and multifocality have major drawbacks, including decreased stereoacuity [5] and contrast sensitivity [6].

Presbyopia laser blended vision (LBV) (Carl Zeiss Meditec) is an EDOF procedure on the cornea, achieved using non-linear aspheric profiles and combined with the micro-monovision (−1.50 D) protocol to treat presbyopia. Using this protocol, the intended postoperative refraction is plano for the dominant eye and in the range of −1.00 to −1.50 D for the nondominant eye [7,8] and implemented into the MEL 80 and MEL 90 platforms (Carl Zeiss Meditec AG). However, the complete assessment of vision-related abilities should consider visual function (the performance of components of the visual system) and functional vision (visual task-related ability) [2]. Typical visual function tests include assessment of visual acuity, contrast sensitivity, visual fields, tests for binocular vision, color, depth, and motion perception, etc. These properties represent an aspect of visual function, each of which may impact an individual's level of functional vision [9] and thus patient satisfaction after a presbyopia correction surgery.

Presbyopia LBV is effective for all types of ametropia, without loss of contrast sensitivity or stereoacuity [10,11], and with high patient satisfaction [12].

The procedure is almost the same as LASIK; it is painless, although a feeling of pressure on the eyes may be noticed. It typically lasts 5-10 minutes for each eye and 10-15 minutes for the whole procedure. Anesthetic eye drops are used before the procedure and help to numb the eyes. After lying down, a device called a lid speculum will be used to keep the eye open and prevent any involuntary blinking. A vacuum ring is also placed on the eye whilst the femtosecond laser is creating the flap. During this time, the vision may appear dimmer and darker. The surgeon will then lift the flap and the exposed cornea will be treated (lasered) with the excimer laser. The flap is then re-positioned, and the same procedure is repeated on the other eye, repeatable if the results are not satisfactory or due to aging [13].

The success of Presbyond is attributed to several key mechanisms. First, the procedure intentionally increases corneal spherical aberration, enhancing the depth of field and enabling clear vision across various distances without the need for glasses. Second, it creates a mildly multifocal epithelial thickness profile beneath the eye's surface, providing multiple focusing points and further improving the range of clear vision. Third, Presbyond capitalizes on pupil constriction during accommodation, utilizing the pinhole effect to enhance the depth of field on the retinal image, contributing to clearer vision at different distances. Additionally, the procedure employs retinal and cortical processing to enhance the contrast of the retinal image, aiding in the integration of visual information and overall vision quality. Furthermore, the intentional imbalance in refractive power, known as anisometropia, is well-tolerated by most patients, resulting in a blend zone and continuous vision from distance to near vision between the eyes. Finally, central cortical processing of the spherically aberrated retinal image, including neuronal gating and blur suppression, allows for simultaneous binocular vision and preserves stereoacuity, distinguishing it from monovision approaches [13].

Patient satisfaction affects clinical outcomes, patient retention, and medical malpractice claims. It affects the timely, efficient, and patient-centered delivery of quality health care. Patient satisfaction is thus a proxy but a very effective indicator to measure the success of doctors and hospitals [14]. Therefore, satisfaction studies can provide important feedback about the quality of care and outcomes, which in turn allows the medical provider to improve the services offered [15]. To date, there have been no studies that have specifically evaluated the quality of life and patient satisfaction after Presbyopia surgery for patients above 40 in Taif City. This study assesses subjective patient satisfaction, self-perceived outcomes, and subjective visual improvement [16].

Null hypothesis

Participants aged 40 years and older will report a satisfaction rate less than or equal to 89% after two months following the procedure.

## Materials and methods

Study design

This was a cross-sectional study with a duration of four years from the first of January 2019 until the first of February 2023

Study settings

We conducted phone interviews to increase the response rate with a prepared questionnaire that was studied to achieve equality between participants to determine whether they were satisfied or not with the results after this surgery. We explained to the participants that their answers were for research purposes, took consent, and assured them that their information would be kept private.

Sampling and population (including inclusion and exclusion criteria)

Patients who underwent the Presbyond surgery in Taif City, Saudi Arabia, and were aged 40 years and older were included in the study.

We excluded any patients who have had the procedure within the past two months of the contact date, any patients who have been clinically diagnosed with any preexisting conditions affecting vision for example DM complications affecting vision, autoimmune conditions affecting vision, congenital diseases affecting vision, recent history of eye trauma, and who end up with complications during the procedure. The follow-up duration ranges from 1 year up to 3 years after the procedure.

The questionnaire included open and closed-end questions, focusing mainly on two time intervals: 1 month and 1 year, and the overall experience.

Tools and data collection procedure

Data was collected from randomly selected patients who had undergone Presbyond surgery in Taif City, Saudi Arabia and were aged 40 years and older. Contact information was obtained solely from hospital records of patients who had undergone Presbyond surgery.

Statistical design

The minimum sample size was eighty-nine patients (48 myopic,41 hyperopic). It was calculated using the 2004 Raosoft sample size calculator with 10% margin of error; we accepted with a 95% confidence level for 20000 population size and 50% response distribution. Data was analyzed using IBM SPSS Statistics for Windows, Version 22 (Released 2013; IBM Corp., Armonk, New York, United States). The means and the standard deviations were calculated for numerical data, while frequencies and percentages were used for categorical data. Differences between groups were tested by statistical tests of significance; t-test, chi-squared test, etc., and the statistical significance level will be set at 5%.

The study had high satisfaction rates. Ethical approval was obtained from Taif University which is accredited by the National Committee for Bioethics (HAO-02-T-105) with application no. 44-084.

## Results

Table 1 shows that the average age of participants in the study was 48.97 years, with a standard deviation of 5.27. Among the participants, 76.4% of participants (n=68) were females and 23.6% (n=21) were males. On average, participants reported having started to notice a decline in their ability to see close objects such as reading and using their phone at the age of 35.270 years old. Furthermore, most of the participants indicated that they learned about refractive surgery, through advertisements (44.9%, n=40) and a friend's recommendation (19.1%, n=17). Few participants learned about refractive surgery from a doctor's recommendation (12.4%, n=11) or through reading (2.2%, n=2) or social media (12.4%, n=11). In terms of satisfaction with the pre-surgery procedures, the majority of participants (64.1%, n=57) reported being very satisfied, followed by 23.6% of participants (n=21) who were satisfied. For those who indicated that they were dissatisfied, the major reasons for the dissatisfaction included delays in the process, dry eyes, high cost, the need to wear glasses even after the procedure, and lack of sufficient education about the procedure and its possible complications.

**Table 1:** General information of the participants (N=89) General information of patients has been presented as (n), (%), mean ± SD.

Variables	Mean ± STD/ n (%)
Gender	
Females	68(76.4%)
Males	21 (23.6%)
Age	48.966 ± 5.27
Have you ever undergone refractive surgery?	
No	0 (0.00%)
Yes	89 (100%)
Before undergoing surgery, at what age did you start to notice a decline in your ability to see close objects such as reading and using your phone?	35.270 ± 12.836
How did you learn about refractive surgery?	
Doctor’s recommendation	11 (12.4%)
Through advertisements	40 (44.9%)
From a friend's recommendation	17 (19.1%)
Reading	2 (2.2%)
Social media	11 (12.4%)
How satisfied are you with the pre-surgery procedures, such as health education, medical testing, and communication with your doctor?	
Very satisfied	57 (64.1%)
Satisfied	21 (23.6%)
Neutral	1 (1.1%)
Dissatisfied	8 (9.0%)
Very dissatisfied	2 (2.2%)

Table 2 shows that most of the participants (89.9%, n=80) did not experience any problems during the refractive surgery, such as delays, longer surgery duration, or pain. In terms of the time, it took for participants to experience complete improvement and return to normal life after surgery, 28.1% (n=25) reported this occurred within 1-30 days, while 39.2% (n=35) experienced it within 1-3 months. After the first month of surgery, 56.2% (n=50) of participants reported being completely satisfied with the results. This level of satisfaction did not change for 25.8% (n=23) of participants after one year of surgery, while 11.2% (n=10) became dissatisfied with the results. For those who were dissatisfied, the major reasons were delay in the process and pain. The goal of the surgery was achieved for 80.9% (n=72) of participants, who reported not needing glasses and experiencing an overall improvement in their quality of life. Eye strain and fatigue during the first 15 minutes of using a phone or reading books were reported by 40.4% (n=36) of participants after surgery. An overall improvement in quality of life, including activities such as reading, using a mobile phone, reading road signs, and watching TV, was reported by 80.9% (n=72) of participants after the surgery. Around 55.1% (n=49) of participants stated that they were very likely to recommend refractive surgery to a family member or friend experiencing vision problems, while 7.9% (n=7) said they were very unlikely to do so. In terms of satisfaction, 47.2% (n=42) of participants reported being very satisfied with their refractive surgery, while 14.6% (n=13) were dissatisfied and 5.6% (n=5) were very dissatisfied. However, for those who reported being dissatisfied, the major reasons were as follows: blurred vision, the need to wear glasses until now, dry eyes, the high expenses, the need to use one eye to look far away and another for looking up close and experiencing pain.

**Table 2:** Assessment of the general characteristics of the participants after laser correction of presbyopia (presbyond) (N=89) Characteristics after surgery has been presented as (n,%)

During the surgery, did you experience any problems such as delay in the surgery date, the surgery taking longer than expected, or you feeling pain during the surgery?	
No	80 (89.9%)
Yes	9 (10.1%)
When did you feel a complete improvement and return to your normal life after surgery?	
Immediately	7 (7.9%)
Never	7 (7.9%)
1-30 Days	25 (28.1%)
1-3 months	35 (39.2%)
4-6 months	11 (12.4%)
7-12 months	4 (4.5%)
After the first month of surgery, are you completely satisfied with the results?	
No	39 (43.8%)
Yes	50 (56.2%)
After one year of surgery, has your level of satisfaction with it changed?	
Yes, I am more satisfied than before	55 (2.4%)
Yes, I am dissatisfied with the results of the surgery	10 (11.2%)
No, it has not changed	23 (25.8%)
Does not apply	1 (1.1%)
Over time, do you feel that the surgery has achieved the goal for which you had the surgery, such as (not needing glasses, the ability to see close objects clearly, and an overall improvement in quality of life)?	
No	17 (19.1%)
Yes	72 (80.9%)
After surgery, do you feel eye strain and fatigue during the first 51 minutes of using your phone or reading books?	
No	53 (59.6%)
Yes	36 (40.4%)
Have you noticed an overall improvement in your quality of life, such as (reading books, using your mobile phone, reading road signs, and watching TV) after having the surgery?	
No	17 (19.1%)
Yes	72 (80.9%)
How likely are you to recommend refractive surgery to a family member or friend who is experiencing vision problems?	
Very likely	49 (55.1%)
Likely	19 (21.3%)
Neutral or I don't know	4 (4.5%)
Not likely	7 (7.9%)
Very unlikely	10 (11.2%)
Overall, how satisfied are you with your refractive surgery?	
Very satisfied	42 (47.2%)
Satisfied	27 (30.3%)
Neutral	2 (2.2%)
Dissatisfied	13 (14.6%)
Very dissatisfied	5 (5.6%)

Table 3 shows that the timing of complete improvement after surgery was significantly associated with the outcomes (p=0.000). Only 2.4% (n=2) reported feeling an immediate improvement, while most of the patients (48.3% n=43) experienced complete improvement within 1-30 days, and 25.8% (n=23) over a longer period within 4-6 months. The perception of achieving the surgical goals was also significantly associated with perceived outcomes (p=0.013). Most of the participants, (80.9%, n=72), felt that the surgery had achieved its intended goals. Furthermore, the presence of eye strain and fatigue during phone use or reading books was also significantly associated with perceived outcomes (p=0.023). Most patients, (59.6%, n=53), did not experience eye strain and fatigue during the first 15 minutes of using their phone or reading books.

**Table 3:** Association between the outcomes and visual improvement and the period after surgery (after one month) The association between the outcomes and visual improvement has been presented as (n), (%). P-value considered significant at p<0.05

		P-Value*
When did you feel a complete improvement and return to your normal life after surgery?	2 (2.4%)	0.000
Immediately	9 (10.1%)	
Never	10 (11.2%)	
1-30 Days	43 (48.3%)	
1-3 months	1 (1.1%)	
4-6 months	23 (25.8%)	
7-12 months	1 (1.1%)	
Over time, do you feel that the surgery has achieved the goal for which you had the surgery, such as (not needing glasses, the ability to see close objects clearly, and an overall improvement in quality of life)?		0.013
No	17 (19.1%)	
Yes	72 (80.9%)	
After surgery, do you feel eye strain and fatigue during the first 15 minutes of using your phone or reading books?		0.023
No	53 (59.6%)	
Yes	36 (40.4%)	
Have you noticed an overall improvement in your quality of life, such as (reading books, using your mobile phone, reading road signs, and watching TV) after having the surgery?		0.013
No	17 (19.1%)	
Yes	72 (80.9%)	

Table 4 shows that there is a significant difference between the level of satisfaction and the period after surgery (after one month). The mean satisfaction score after one month of surgery is 2.494 units higher than the mean satisfaction score before surgery. This difference is statistically significant (p < .001).

**Table 4:** Difference between groups’ level of satisfaction and the period after surgery (after one month) Various statistical tests were presented. P considered statistically significant at p<0.05

		Paired Differences					t	df	Sig. (two-tailed)
		Mean	Std. Deviation	Std. Error Mean	95% Confidence Interval of the Difference				
					Lower	Upper			
Pair 1	Satisfaction - Period	2.494	1.035	.110	2.276	2.712	22.739	88	.000

## Discussion

The study found that the average age of participants was 48.97 years, indicating that most of the participants were middle-aged. This suggests that refractive surgery is sought after by individuals in their late forties who may be experiencing age-related vision problems.

The majority of participants in the study were females, accounting for 76.4% (n=68) of the sample. This could suggest that women are more likely to seek out refractive surgery or that they are more proactive in addressing their vision issues. Participants reported starting to notice a decline in their ability to see close objects, such as reading or using their phone, at an average age of 35.270 years. This supports previous research indicating that presbyopia, the loss of near vision with age, typically begins in the early to mid-40s. Many participants learned about refractive surgery through advertisements (44.9%, n=40) and a friend's recommendation (19.1%, n=17). This highlights the importance of advertising and word-of-mouth in informing individuals about available treatment options for vision problems. In terms of satisfaction with the pre-surgery procedures, the majority of participants (64.1%, n=57) reported being very satisfied. This indicates that most individuals had a positive experience leading up to the surgery, which may have contributed to their decision to undergo the procedure. For those who indicated dissatisfaction, the main reasons cited included delays in the process, dry eyes, the cost of the surgery, the continued need for glasses, and a lack of sufficient education about the procedure. These findings suggest that improvements could be made in the pre-surgery process, including better communication and education for patients, as well as addressing potential side effects such as dry eyes. These findings were in line with the finding from the study by Kohnen et al., which showed that the aspheric multifocal LASIK procedure provided excellent uncorrected near visual acuity and high patient satisfaction. The majority of patients reported good vision without glasses for near activities. The study suggests that aspheric multifocal LASIK is an effective option for presbyopic patients seeking independence from glasses for near vision [17].

According to the findings of the study, the majority of participants did not experience any problems during their refractive surgery, such as delays, longer surgery duration, or pain. This is a positive outcome, as it indicates that the surgical procedure went smoothly for most individuals. In terms of the recovery process, a considerable number of participants (28.1%, n=25) reported experiencing complete improvement and returning to normal life within 1-30 days after surgery. A slightly larger percentage (39.2%, n=35) experienced this within 1-3 months. This suggests that the recovery time can vary among individuals, but the majority of participants were able to resume their normal activities within a relatively brief period. After the first month of surgery, more than half of the participants (56.2%, n=50) reported being completely satisfied with the results. This level of satisfaction remained consistent for a quarter of participants after one year, while a smaller percentage (11.2%, n=10) became dissatisfied with the results. The main reasons for dissatisfaction were delays in the process and ongoing pain. Overall, the goal of the surgery was achieved for the majority of participants (80.9%, n=72), who no longer needed glasses and experienced an improvement in their quality of life. However, some participants (40.4%, n=36) reported experiencing eye strain and fatigue during the first 15 minutes of using a phone or reading books after the surgery. This suggests that there may be some short-term discomfort or adjustment period following the surgery. This concurs with Russo and his colleagues’ study findings which noted that PRESBYOND provided good uncorrected visual acuity at all distances, high patient satisfaction, and improved near vision in presbyopic patients. Additionally, the treatment was safe and effective for both myopic and hyperopic patients, with no significant changes in distance vision or contrast sensitivity [18].

Despite these potential minor issues, the majority of participants (80.9%, n=72) reported an overall improvement in their quality of life after the surgery. This included activities such as reading, using a mobile phone, reading road signs, and watching TV. This indicates that the surgery had a positive impact on their daily lives and activities. In terms of recommendations, more than half of the participants (55.1%, n=49) stated that they were very likely to recommend refractive surgery to a family member or friend experiencing vision problems. This highlights a high level of satisfaction and confidence in the procedure. Overall, nearly half of the participants (47.2%, n=42) reported being very satisfied with their refractive surgery. However, a small percentage (14.6%, n=13) expressed dissatisfaction, and an even smaller percentage (5.6%, n=5) were very dissatisfied. The major reasons for dissatisfaction included blurred vision, the need to still wear glasses, dry eyes, the cost of the surgery, and the need to use one eye for near vision and another for distance vision. These findings suggest that there is room for improvement in terms of addressing these concerns. The findings were consistent with the findings reported by Ganesh et al. who established that showed that laser-blended vision with non-linear aspheric micro-monovision is an effective treatment option for presbyopia and myopia or hyperopia with a satisfactory visual outcome and high patient satisfaction. There was a significant improvement in near, intermediate, and distance vision, and no serious complications were reported [12].

In addition, the study found that the mean satisfaction score after one month of surgery is 2.494 units higher than the mean satisfaction score before surgery. This difference is statistically significant (p < .001). Therefore, patients tend to be more satisfied with their surgery outcome after a month compared to before the surgery. The timing of complete improvement after surgery was found to be significantly associated with the outcomes, with a p-value of 0.000. This means that the timing of complete improvement has a strong relationship with how patients perceive the outcomes of the surgery. A small percentage of patients (2.4%, n=2) reported feeling an immediate improvement after surgery, while the majority experienced complete improvement within a specific period. This includes 48.3% (n=43) who reported complete improvement within 1-30 days and 25.8% (n=23) over a longer period within 4-6 months. The perception of achieving the surgical goals was also found to be significantly associated with perceived outcomes, with a p-value of 0.013. This means that patients who felt that the surgery had achieved its intended goals were more likely to have positive outcomes. The presence of eye strain and fatigue during phone use or reading books was also found to be significantly associated with perceived outcomes, with a p-value of 0.023. This means that patients who did not experience eye strain and fatigue during these activities were more likely to have positive outcomes after surgery. This complements the finding in a study by Luger et al. which found that patients experienced significant improvements in distance and near visual acuity, with stable outcomes up to three years post-surgery. The procedure was also found to be safe, with no significant adverse events observed [19].

Some limitations of the study include the small sample size, which may limit the generalizability of the findings. Additionally, the study relied on self-reported data, which may be subject to recall bias or misinterpretation. The study also did not assess the specific type of refractive surgery that participants underwent, which may have affected their experiences and satisfaction. Finally, the study did not include a control group, making it difficult to determine the true impact of the surgery on participants' outcomes and satisfaction.

## Conclusions

In conclusion, this study delves into the popularity of refractive surgery among middle-aged individuals and above with age-related vision issues. The study indicates that these vision problems are typically noticed around the age of 35. Pre-surgery experiences were satisfactory, but communication and patient education could be enhanced. Most of the participants asserted they had smooth surgeries and quick post-operative recoveries, leading to an overall improvement in their quality of life. However, some experienced short-term discomfort and adjustment periods. Patient satisfaction significantly increased one month after surgery, with timing and goal achievement playing vital roles. Eye strain and fatigue during specific activities were also associated with outcomes.

To improve patient experiences, healthcare providers and refractive surgeons should focus on clearer communication about the surgery's risks, benefits, and potential side effects. Addressing common concerns, like blurred vision, glasses reliance, and dry eyes, should be a priority. Additionally, providing support and resources for patients to manage post-operative discomfort and vision changes can lead to better satisfaction and outcomes.

## Appendices

Interview questions

We conducted phone interviews with a prepared questionnaire, as follows:

Do you agree to participate in this survey? 

- Yes

- No

Sex?

- Male

-Female

Age?

Have you ever undergone vision correction surgery?

- Yes

- No

When did you have your vision correction surgery?

Before the operation, at what age did you begin to notice a weakening of the eye’s ability to see nearby objects? (Reading and using the phone)?

How did you know about the vision correction process?

- Through advertisements

- Recommended by a friend

- Reading

- Social media programs

How satisfied are you with the pre-operative preparation procedures ( health education, medical examinations, and the doctor’s communication with you)?

- Very satisfied

- Satisfied

- Neutral

- Not satisfied

- Never satisfied

If your answer to the previous question is (not satisfied), please state the reason for your dissatisfaction

During the procedure, did you encounter any problems, such as (a delay in the procedure date, the operation took longer than expected of you, you felt pain during the operation )

- Yes

- No

If your answer to the previous question is (yes ), please mention the problems you encountered during the procedure

When did you feel completely better and return to normal life after the operation?

After the first month of the operation, do you feel completely satisfied with its results?

- Yes

- No

One year after the operation, has your satisfaction with it changed?

- Yes, I am more satisfied than before

- Yes, I have become dissatisfied with the results of the operation

- No, it hasn't changed

-do not apply

Over time, do you feel that the operation achieved the goal for which you performed the operation, such as (getting rid of glasses, being able to see close objects clearly, improving the quality of life in general)?

- Yes

- No

- Other than that

After the procedure, do you feel eye strain and fatigue while using the phone or reading books for the first 15 minutes?

- Yes

- No

Did you notice a general improvement in your quality of life (such as reading books, using your mobile phone, reading road signs, and watching TV) after your procedure?

- Yes

- No

How likely are you to recommend vision correction surgery to one of your relatives or friends who suffer from vision problems?

- Very likely

- Probably

- Neutral or I don't know

- Not likely

- It is very unlikely

In general, how satisfied are you with your vision correction process?

- Very satisfied

- Satisfied

- Neutral

- Not satisfied

- Never satisfied

If your answer to the previous question is (not satisfied), please state the reason for your dissatisfaction

## References

[1] Mathis T, Jardel P, Loria O (2019). New concepts in the diagnosis and management of choroidal metastases. Prog Retin Eye Res.

[2] Bennett CR, Bex PJ, Bauer CM, Merabet LB (2019). The assessment of visual function and functional vision. Semin Pediatr Neurol.

[3] Koopmans SA, Terwee T, Barkhof J, Haitjema HJ, Kooijman AC (2003). Polymer refilling of presbyopic human lenses in vitro restores the ability to undergo accommodative changes. Invest Ophthalmol Vis Sci.

[4] Nishi Y, Mireskandari K, Khaw P, Findl O (2009). Lens refilling to restore accommodation. J Cataract Refract Surg.

[5] Fawcett SL, Herman WK, Alfieri CD, Castleberry KA, Parks MM, Birch EE (2001). Stereoacuity and foveal fusion in adults with long-standing surgical monovision. J AAPOS.

[6] Tanabe H, Tabuchi H, Shojo T, Yamauchi T, Takase K (2020). Comparison of visual performance between monofocal and multifocal intraocular lenses of the same material and basic design. Sci Rep.

[7] Reinstein DZ, Carp GI, Archer TJ, Gobbe M (2012). LASIK for presbyopia correction in emmetropic patients using aspheric ablation profiles and a micro-monovision protocol with the Carl Zeiss Meditec MEL 80 and VisuMax. J Refract Surg.

[8] Reinstein DZ, Archer TJ, Gobbe M (2011). LASIK for myopic astigmatism and presbyopia using non-linear aspheric micro-monovision with the Carl Zeiss Meditec MEL 80 Platform. J Refract Surg.

[9] Lennie P, Van Hemel SB (2002). Visual Impairments: Determining Eligibility for Social Security Benefits.

[10] Zhang T, Sun Y, Weng S (2016). Aspheric micro-monovision LASIK in correction of presbyopia and myopic astigmatism: early clinical outcomes in a Chinese population. J Refract Surg.

[11] Liu XX, Teng YF, Gao M, Liang XD, Yu YP, Liu W (2018). The optic nerve head perfusion and its correlation with the macular blood perfusion in unilateral idiopathic macular hole: an optical coherence tomography angiography study. Int J Ophthalmol.

[12] Ganesh S, Brar S, Gautam M, Sriprakash K (2020). Visual and refractive outcomes following laser blended vision using non-linear Aspheric micro-monovision. J Refract Surg.

[13] Reinstein DZ, Archer TJ, Gobbe ME Aspheric Ablation Profile for Presbyopic Corneal Treatment using the MEL80 and CRS Master Laser Blended Vision Module 2011. https://api.semanticscholar.org/CorpusID:13807789.

[14] Prakash B (2010). Patient satisfaction. J Cutan Aesthet Surg.

[15] Hill JC (2002). An informal satisfaction survey of 200 patients after laser in situ keratomileusis. J Refract Surg.

[16] Bamashmus MA, Hubaish K, Alawad M, Alakhlee H (2015). Functional outcome and patient satisfaction after laser in situ keratomileusis for correction of myopia and myopic astigmatism. Middle East Afr J Ophthalmol.

[17] Kohnen T, Böhm M, Herzog M, Hemkeppler E, Petermann K, Lwowski C (2020). Near visual acuity and patient-reported outcomes in presbyopic patients after bilateral multifocal aspheric laser in situ keratomileusis excimer laser surgery. J Cataract Refract Surg.

[18] Russo A, Reinstein DZ, Filini O (2022). Visual and refractive outcomes following laser blended vision with non-linear aspheric micro-anisometropia (PRESBYOND) in myopic and hyperopic patients. J Refract Surg.

[19] Luger MH, McAlinden C, Buckhurst PJ, Wolffsohn JS, Verma S, Arba-Mosquera S (2020). Long-term outcomes after LASIK using a hybrid bi-aspheric micro-monovision ablation profile for presbyopia correction. J Refract Surg.

